# Parameter subset reduction for imaging-based digital twin generation of patients with left ventricular mechanical discoordination

**DOI:** 10.1186/s12938-024-01232-0

**Published:** 2024-05-13

**Authors:** Tijmen Koopsen, Nick van Osta, Tim van Loon, Roel Meiburg, Wouter Huberts, Ahmed S. Beela, Feddo P. Kirkels, Bas R. van Klarenbosch, Arco J. Teske, Maarten J. Cramer, Geertruida P. Bijvoet, Antonius van Stipdonk, Kevin Vernooy, Tammo Delhaas, Joost Lumens

**Affiliations:** 1 Department of Biomedical Engineering, Cardiovascular Research Institute Maastricht (CARIM), Maastricht University, Maastricht, The Netherlands; 2 Group SIMBIOTX, Institut de Recherche en Informatique et en Automatique (INRIA), Paris, France; 3 Department of Biomedical Engineering, Eindhoven University of Technology, Eindhoven, The Netherlands; 4 Department of Cardiology, Suez Canal University, Ismailia, Egypt; 5 Division of Heart and Lungs, Department of Cardiology, University Medical Center Utrecht (UMCU), Utrecht, The Netherlands; 6 Department of Cardiology, Cardiovascular Research Institute Maastricht (CARIM), Maastricht University, Maastricht, The Netherlands; 7 Department of Cardiology, Maastricht University Medical Center (MUMC), Maastricht, The Netherlands; 8 Department of Cardiology, Radboud University Medical Center, Nijmegen, The Netherlands

**Keywords:** Digital twin, Mechanical discoordination, Left bundle branch block, Myocardial infarction, Myocardial strain, Sensitivity analysis, Identifiability analysis, Parameter estimation, Myocardial work, Disease characterization

## Abstract

**Background:**

Integration of a patient’s non-invasive imaging data in a digital twin (DT) of the heart can provide valuable insight into the myocardial disease substrates underlying left ventricular (LV) mechanical discoordination. However, when generating a DT, model parameters should be identifiable to obtain robust parameter estimations. In this study, we used the CircAdapt model of the human heart and circulation to find a subset of parameters which were identifiable from LV cavity volume and regional strain measurements of patients with different substrates of left bundle branch block (LBBB) and myocardial infarction (MI). To this end, we included seven patients with heart failure with reduced ejection fraction (HFrEF) and LBBB (study ID: 2018-0863, registration date: 2019–10–07), of which four were non-ischemic (LBBB-only) and three had previous MI (LBBB-MI), and six narrow QRS patients with MI (MI-only) (study ID: NL45241.041.13, registration date: 2013–11–12). Morris screening method (MSM) was applied first to find parameters which were important for LV volume, regional strain, and strain rate indices. Second, this parameter subset was iteratively reduced based on parameter identifiability and reproducibility. Parameter identifiability was based on the diaphony calculated from quasi-Monte Carlo simulations and reproducibility was based on the intraclass correlation coefficient (${ICC}$) obtained from repeated parameter estimation using dynamic multi-swarm particle swarm optimization. Goodness-of-fit was defined as the mean squared error (${{{\chi}}}^{{2}}$) of LV myocardial strain, strain rate, and cavity volume.

**Results:**

A subset of 270 parameters remained after MSM which produced high-quality DTs of all patients (${{{\chi}}}^{{2}}$ < 1.6), but minimum parameter reproducibility was poor (${{ICC}}_{{min}}$ = 0.01). Iterative reduction yielded a reproducible (${{ICC}}_{{min}}$ = 0.83) subset of 75 parameters, including cardiac output, global LV activation duration, regional mechanical activation delay, and regional LV myocardial constitutive properties. This reduced subset produced patient-resembling DTs (${{{\chi}}}^{{2}}$ < 2.2), while septal-to-lateral wall workload imbalance was higher for the LBBB-only DTs than for the MI-only DTs (*p* < 0.05).

**Conclusions:**

By applying sensitivity and identifiability analysis, we successfully determined a parameter subset of the CircAdapt model which can be used to generate imaging-based DTs of patients with LV mechanical discoordination. Parameters were reproducibly estimated using particle swarm optimization, and derived LV myocardial work distribution was representative for the patient’s underlying disease substrate. This DT technology enables patient-specific substrate characterization and can potentially be used to support clinical decision making.

**Supplementary Information:**

The online version contains supplementary material available at 10.1186/s12938-024-01232-0.

## Background

Left ventricular (LV) mechanical discoordination is defined by the reciprocal shortening and stretching of myocardium within the LV wall [1–3]. Myocardial stretching during systole involves wasted work and is, therefore, detrimental for cardiac pump function [4]. Mechanical discoordination is often caused by an electrical conduction disturbance, such as left bundle branch block (LBBB), but it can also be induced by myocardial ischemia or infarction [2]. The underlying disease substrates of mechanical discoordination determine its progression and the potential effects of cardiac resynchronization therapy (CRT) [2, 5].

To increase insight into a patient’s underlying disease substrates of mechanical discoordination, a digital twin (DT) of the patient’s heart can be developed by integrating patient-specific measurements into a biophysical computational model [6]. Previous studies have demonstrated that LV regional strain measurements reveal different characteristic patterns in hearts with LBBB [7, 8] and myocardial infarction (MI) [9], as well as in hearts with combined LBBB-MI substrates [5, 10]. Myocardial strain measurements are, therefore, valuable to generate a DT. Furthermore, supplementing strain with LV cavity volume measurements, which are derived from routine non-invasive imaging, provides more information on LV systolic and diastolic function.

The CircAdapt model of the human heart and circulation [11] is a suitable model to generate DTs based on these non-invasive imaging data. CircAdapt has previously been shown to realistically simulate myocardial mechanics during LBBB and MI [12, 13]. Furthermore, the model is able to simulate regional mechanics at the same spatial scale as current non-invasive strain imaging technologies and at low computational cost.

However, personalization of CircAdapt is challenged by its large number of model parameters, of which only a subset is identifiable from LV cavity volume and regional strain measurements. Determining these identifiable parameters is important to ensure robust parameter estimations of the DT [14]. To personalize a cardiovascular model with many parameters, several techniques of sensitivity and identifiability analysis have been described before [15, 16]. Van Osta et al. [17] combined these techniques into a framework of parameter subset reduction, which consisted of Morris Screening Method (MSM) [18], quasi-Monte Carlo sampling, and particle swarm optimization (PSO) [19, 20].

In the current study, we utilize and expand upon this framework of parameter subset reduction to determine a subset of identifiable parameters which can be used to generate DTs of patients with LV mechanical discoordination based on their LV cavity volume and regional strain measurements. We also assess the credibility of these DTs by comparing their LV myocardial work distribution against the patient’s electrocardiographic characteristics and location of MI.

## Results

### Sensitivity analysis

Seven consecutive iterations of MSM were performed using a 6-segment LV model, with the last iteration including a subset of important parameters only. Computational time was 4.6 ± 2.7 h per MSM iteration with an average of 2143 ± 1069 trajectories. The results of the first and last iteration are shown in Additional file 1: Figures S1 and S2. The final subset after MSM consisted of $D=108$ parameters, of which ${D}_{{\text{MT}}}=94$ were myocardial tissue parameters, subdivided over the LV (${D}_{{\text{MT}},{\text{LV}}}=84$), left atrium (${D}_{{\text{MT}},{\text{LA}}}=7$) and right ventricle (${D}_{{\text{MT}},{\text{RV}}}=3$). The other 14 parameters included general hemodynamic parameters (${D}_{{\text{GH}}}=3$), pulmonary and systemic circulation parameters (${D}_{{\text{PS}}}=3$), and mechanical activation parameters (${D}_{{\text{MA}}}=8$). Success rates of evaluated trajectories in all iterations were 11.4%, 13.0%, 18.9%, 18.9%, 20.2%, 19.7%, and 20.6%, respectively.

### Parameter estimation and identifiability analysis

**Fig. 1:**
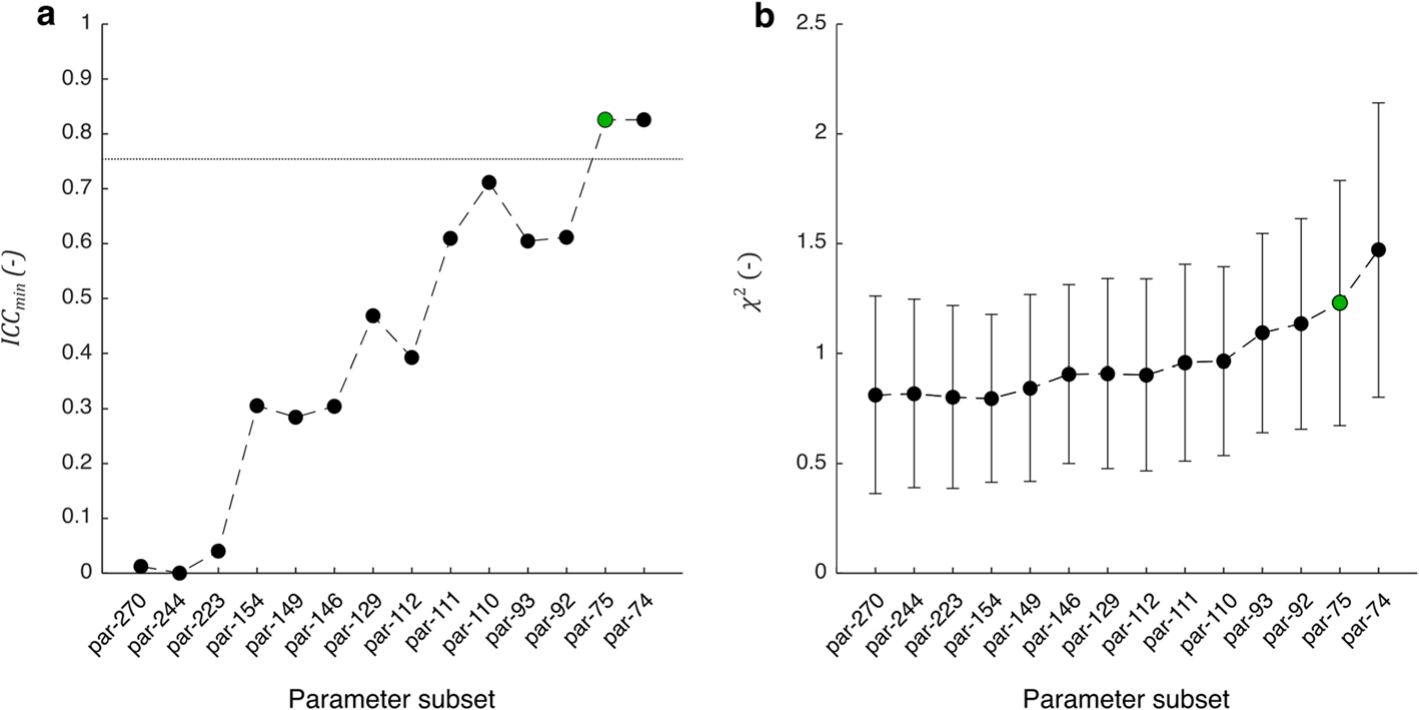
Propagation of **a** minimum intraclass correlation coefficient (${{\text{ICC}}}_{{\text{min}}}$) of estimated model parameters versus **b** mean and standard deviation of cost function value (${\chi }^{2}$) of the population while performing iterations of parameter subset reduction. The green dot (par-75) indicates the parameter subset which was most extensive while satisfying the criterium of ${{\text{ICC}}}_{{\text{min}}}$ > 0.75

The transition from a 6-segment LV model, which was used for the sensitivity and identifiability analyses, to an 18-segment LV model, which was used for patient-specific parameter estimation (PE), resulted in a threefold increase of the number of segmental model parameters. As a result, the total number of model parameters increased from $D=108$ to $D=270$ (par-270; Additional file 2: Table S1). Figure 1a, b demonstrates the propagation of ${{\text{ICC}}}_{{\text{min}}}$ vs. mean and standard deviation of ${\chi }^{2}$ of the patient population while performing iterations of parameter subset reduction. The parameters included in all different subsets evaluated are shown in Additional file 3: Table S2. After twelve iterations of subset reduction, all parameters in the subset satisfied the criterium for reproducibility (${{\text{ICC}}}_{{\text{min}}}=0.83$). This final subset (par-75) consisted of cardiac output ($q0$), atrioventricular delay (${\text{dTauAv}}$), global LV activation duration (${{\text{ADO}}}_{{\text{LV}}}$), and four regional parameters for each of the 18 LV segments: mechanical activation delay (${\text{d}}T$), reference wall area (${\text{AmRef}}$), zero-passive stress sarcomere length (${\text{Ls}}0{\text{Pas}}$), and stiffness coefficient ($k1$).

While ${{\text{ICC}}}_{{\text{min}}}$ increased with reducing parameter subset size, goodness-of-fit decreased as reflected by an increase of the mean ${\chi }^{2}$ of the population from 0.81 ± 0.45 (par-270) to 1.23 ± 0.56 (par-75). One additional iteration of parameter subset reduction (par-74) demonstrated that ${\chi }^{2}$ increased to 1.47 ± 0.67, while ${{\text{ICC}}}_{{\text{min}}}$ remained similar (${{\text{ICC}}}_{{\text{min}}}=0.83$).

**Fig. 2:**
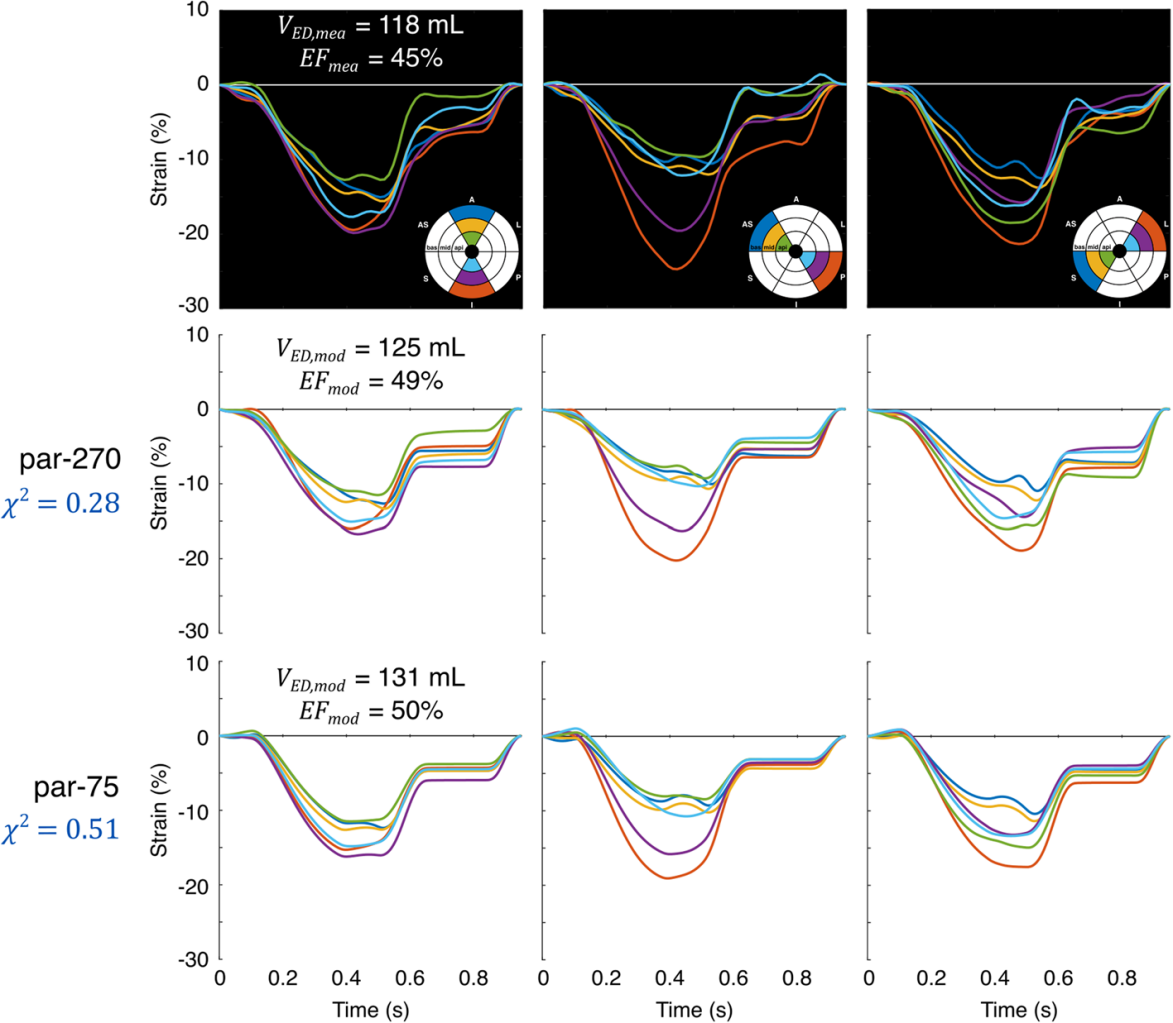
Best fit of par-75 in the patient population according to ${\chi }^{2}$. The upper panels show the echocardiographic strain and volume measurements, while the middle and lower panels show the strains and volumes of the DT for par-270 and par-75, respectively

**Fig. 3:**
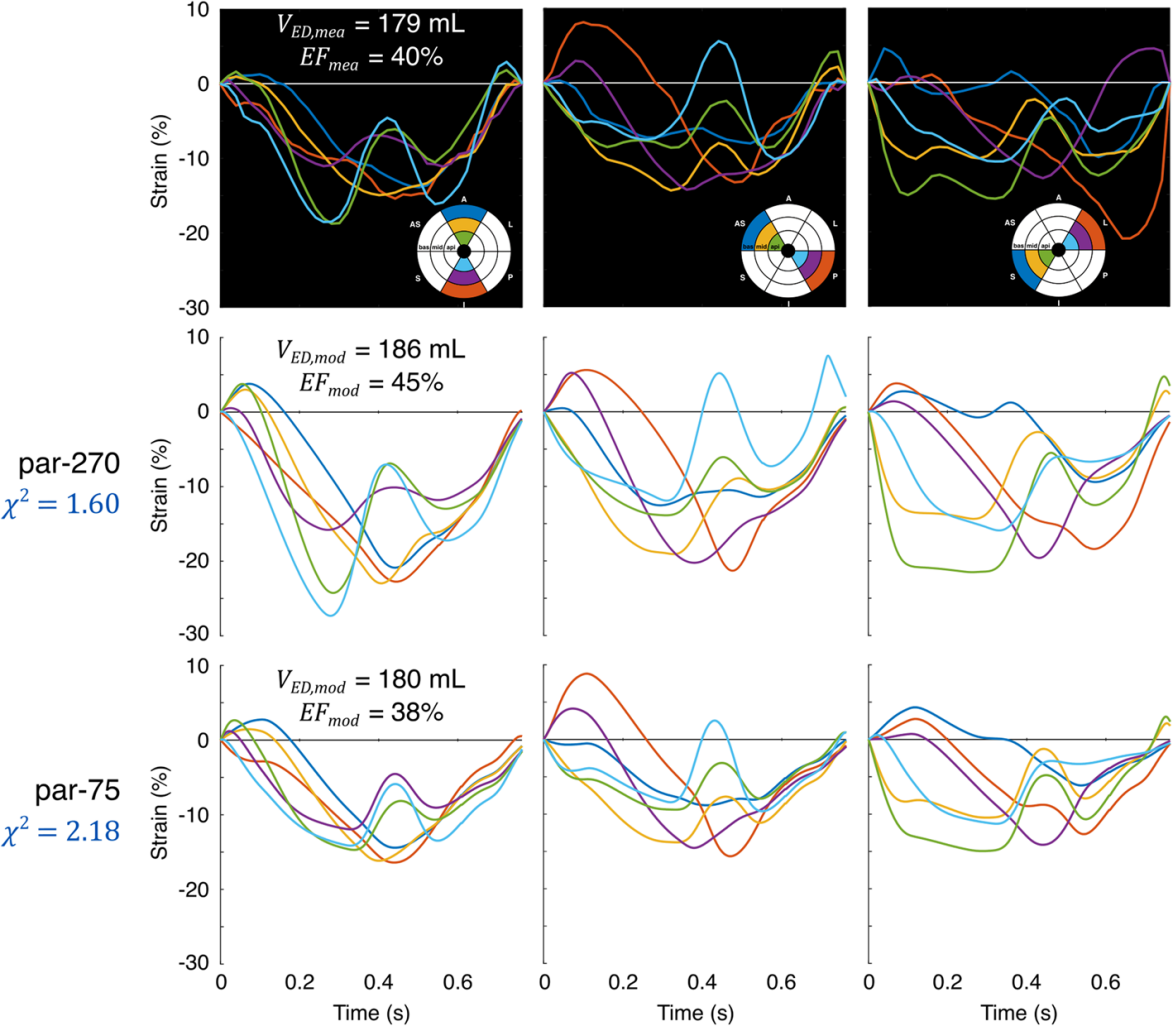
Least good fit of par-75 in the patient population according to ${\chi }^{2}$. The upper panels show the echocardiographic strain and volume measurements, while the middle and lower panels show the strains and volumes of the DT for par-270 and par-75, respectively

Obtained ${\chi }^{2}$ values including the different contributors of ${\chi }^{2}$ for all DTs generated using par-75 are shown in Table 1. Out of all 13 DTs, Figs. 2 and 3 show the best and least good DT according to ${\chi }^{2}$, respectively. The least good DT (LBBB-only patient 2) visually agreed well with the strain measurements.

**Table 1:** Results of parameter estimation using the final parameter subset (par-75) for all 13 digital twins generated

Patient subgroup	Patient number	${\chi }^{2}$ (–)	${V}_{{\text{ED}},{\text{mea}}}$ (mL)	${{\text{EF}}}_{{\text{mea}}}$ (%)	${\chi }_{{V}_{{\text{ED}}}}^{2}$ (–)	${\chi }_{{\text{EF}}}^{2}$ (–)	${\chi }_{\varepsilon }^{2}$ (–)	${\chi }_{\dot{\varepsilon }}^{2}$ (–)
MI-only	1	0.57	132	49	< 0.1	0.3	3.8	17.6
	2	1.06	69	53	2.7	0.6	6.6	30.5
	3	0.64	95	49	1.0	1.9	3.7	17.8
	4	1.47	69	54	0.2	0.5	9.0	46.1
	5	1.12	102	52	0.8	0.6	8.2	32.8
	6	0.51	118	45	0.7	0.5	4.0	14.1
LBBB-only	1	1.22	147	30	< 0.1	0.1	9.9	36.5
	2	2.18	179	40	< 0.1	0.1	18.3	64.5
	3	1.09	190	26	< 0.1	0.3	6.8	34.2
	4	0.95	200	27	< 0.1	< 0.1	7.7	28.6
LBBB-MI	1	1.10	122	35	0.4	0.8	6.0	34.4
	2	2.09	230	27	1.7	0.8	19.6	57.5
	3	1.98	94	47	1.2	0.4	13.1	60.7

${\chi }^{2}$: total cost function value; ${V}_{{\text{ED}},{\text{mea}}}$: measured LV end-diastolic volume; ${{\text{EF}}}_{{\text{mea}}}$: measured LV ejection fraction; ${\chi }_{{V}_{{\text{ED}}}}^{2}$: LV end-diastolic volume contributor; ${\chi }_{{\text{EF}}}^{2}$: LV ejection fraction contributor; ${\chi }_{\varepsilon }^{2}$: strain contributor; ${\chi }_{\dot{\varepsilon }}^{2}$: strain rate contributor

### Digital twin credibility evaluation

**Fig. 4:**
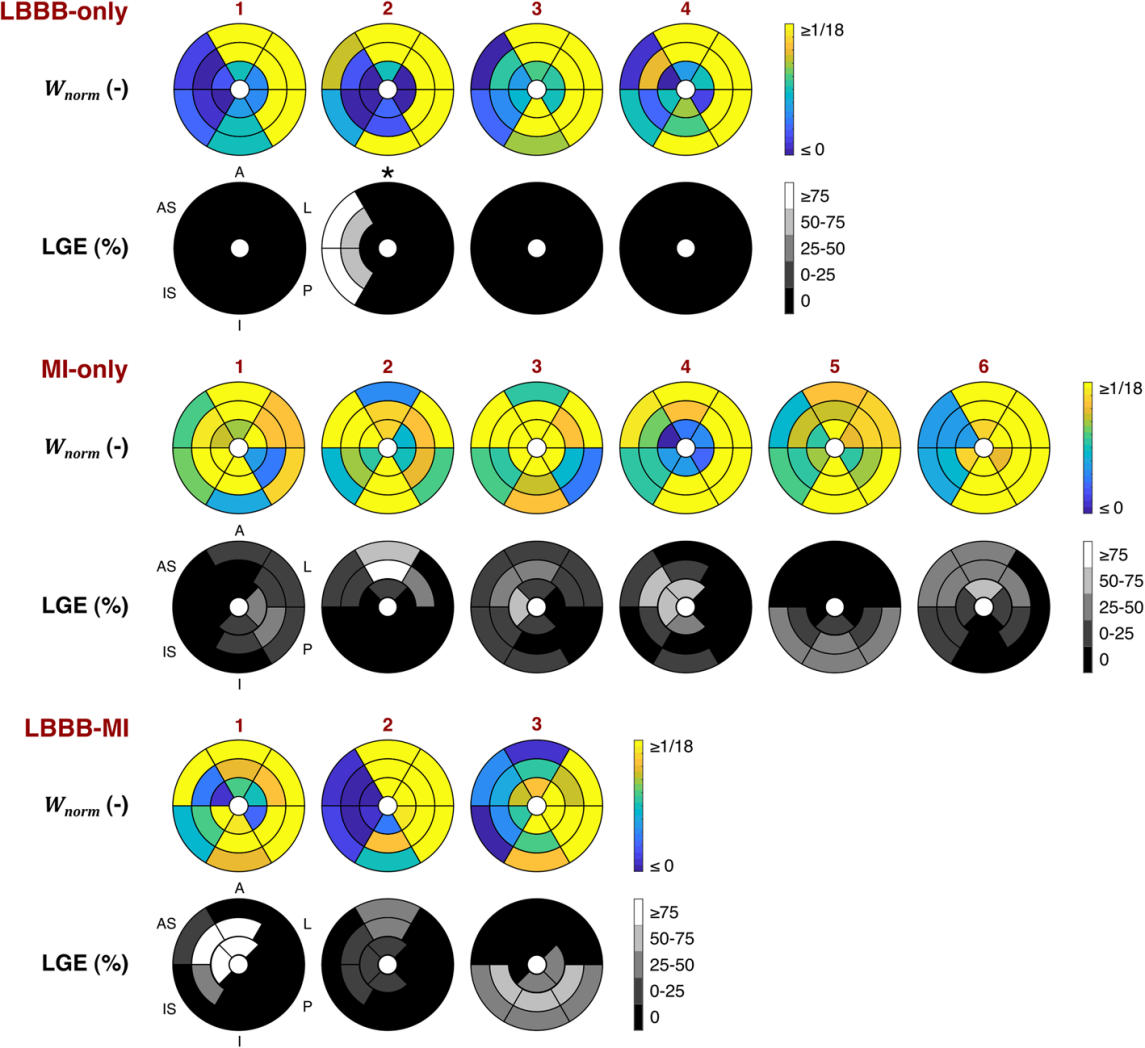
Regional LV normalized work (${W}_{{\text{norm}},i}$) of all DTs generated using par-75, and segmental late gadolinium enhancement (LGE) percentage as quantified using cardiac magnetic resonance imaging. All LBBB-only and LBBB-MI DTs demonstrated a larger amount of work performed by the lateral wall as compared to the septum. This typical pattern of septal-to-lateral wall workload imbalance was not observed in the MI-only DTs. In LBBB-MI DT 1 and in MI-only DTs 1,2, 4 and 6, regions of increased LGE demonstrated reduced ${W}_{{\text{norm}},i}$. *LGE pattern was interpreted as non-ischemic by an experienced cardiologist

**Fig. 5:**
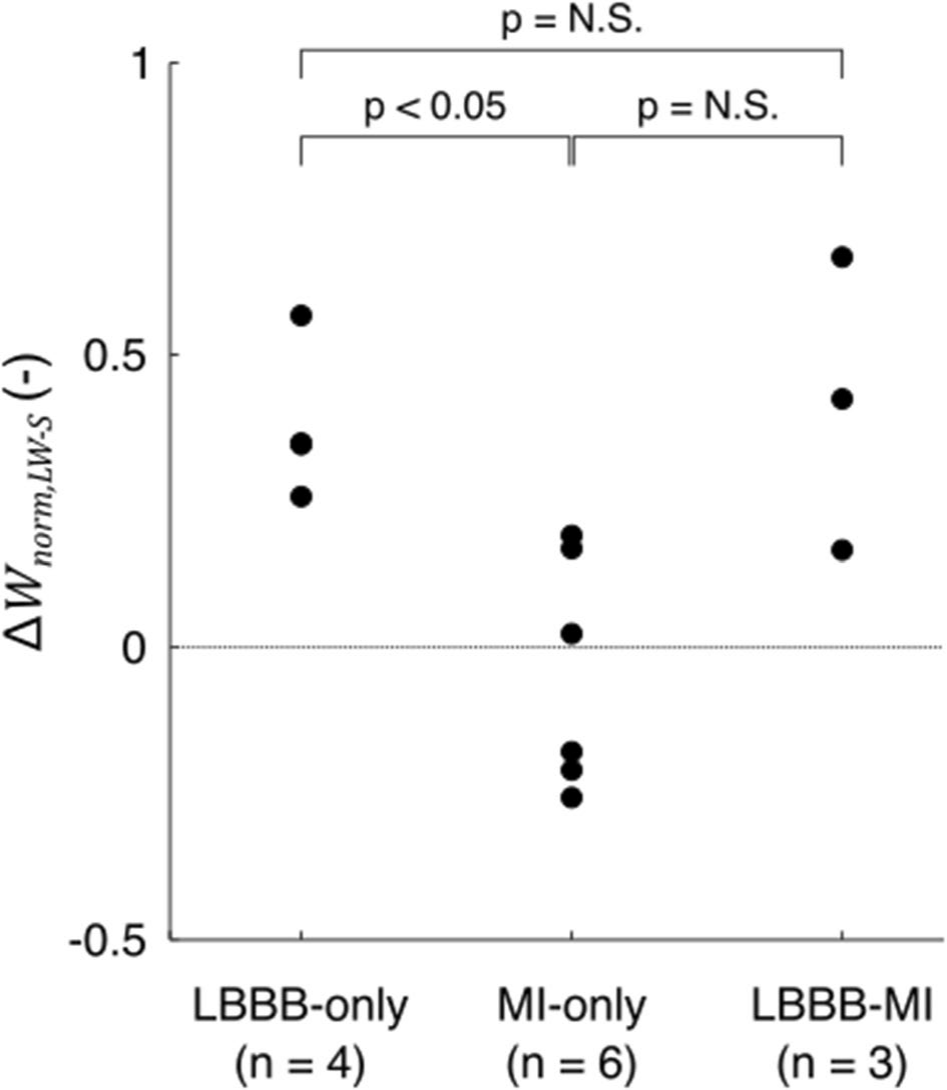
Normalized septal-to-lateral wall workload imbalance ($\Delta {W}_{{\text{norm}},{\text{LW}}-S}$) for the DTs of the three patient subgroups generated using the final subset (par-75). Workload imbalance was significantly higher for the LBBB-only DTs than for the MI-only DTs (*p* < 0.05) but there was no significant difference between LBBB-only and LBBB-MI DTs. While $\Delta {W}_{{\text{norm}},{\text{LW}}-S}$ of LBBB-MI DTs also seemed to be higher than that of MI-only DTs, there was no significant difference between these two subgroups

All LBBB-only DTs demonstrated an increased workload on the LV free wall as compared to the septum (Fig. 4). LBBB-MI DTs 2 and 3 showed a similar characteristic LV workload pattern which was relatively unaffected by their MI substrates. In the DT of patient 1, however, LV workload showed to be more homogeneous. There was no consistent pattern of septal-to-lateral workload imbalance in the MI-only DTs. However, in MI-only DTs 1, 2, 4 and 6, segments with increased LGE showed reduced normalized work (${W}_{{\text{norm}},i}$). Septal-to-lateral work difference ${\Delta W}_{{\text{norm}},{\text{LW}}-S}$ (Fig. 5) was positive in all LBBB-only and LBBB-MI DTs, indicating that more work was performed by the lateral wall than by the septum. In the MI-only group, both positive and negative values of ${\Delta W}_{{\text{norm}},{\text{LW}}-S}$ were found, reflecting a non-consistent septal-to-lateral pattern of workload imbalance. Comparison between subgroups revealed that ${\Delta W}_{{\text{norm}},{\text{LW}}-S}$ was higher for the LBBB-only DTs than for the MI-only DTs (*p* < 0.05). Average $\Delta {W}_{{\text{norm}},{\text{LW}}-S}$ of LBBB-MI DTs also seemed to be higher than that of MI-only DTs, however, no significant difference between these groups was found.

## Discussion

We utilized and expanded upon an existing framework for parameter subset reduction to determine a subset of parameters of the CircAdapt model which were identifiable from LV cavity volume and regional strain measurements of patients with LV mechanical discoordination. The obtained parameter subset included cardiac output, global LV activation duration, regional mechanical activation delay, and regional LV myocardial constitutive properties. This subset was used to personalize the CircAdapt model, resulting in the generation of a digital twin (DT). We created DTs of LBBB patients with and without MI and of narrow-QRS patients with MI. All DTs had similar LV cavity volumes and regional strain patterns as measured in the patients. Moreover, LV myocardial work distribution of the DT was found to provide insight into the patient’s underlying myocardial disease substrate. We hypothesize that the proposed DT methodology can serve as a clinical support tool in patients with LV mechanical discoordination.

The complexity of the cardiovascular system provides a challenge for creating DTs of the heart and circulation, especially since available measurement data is often practically limited [14]. Complexity of developed cardiovascular models is highly variable and depends on the level of detail included to describe cardiac geometry, electromechanics, and hemodynamics [21]. When model complexity is too high for personalization, a reduced-order model can provide a useful alternative. For example, models with lower dimensionality can be developed [22, 23], or models can be reparameterized to reduce their number of parameters [24]. During our iterations of parameter subset reduction, we simplified the CircAdapt MultiPatch model by grouping regional LV parameters into global LV parameters, thereby simulating homogeneity of all LV wall segments. The observed non-continuous increase of ${\text{ICC}}$ (Fig. 1a) showed that identifiability of these global parameters differed from their regional parameter definitions.

The methods which we used for sensitivity and identifiability analysis were chosen based on their strengths for our optimization problem as well as on their computational feasibility [15], but they are not the only techniques to determine parameter sensitivity and identifiability. While we applied MSM, which has previously been used for sensitivity analysis in cardiovascular models with many parameters [16, 25], variance-based sensitivity analysis [15] and polynomial chaos expansion [16, 26] provide powerful methods for lower-dimensional problems. Furthermore, we used diaphony as an index of identifiability, but other identifiability analysis methods have previously been described, such as profile likelihood analysis [27, 28]. This method was considered too computationally demanding, but might provide more insight into the robustness of the final parameter subset. To allow more extensive sensitivity and identifiability analyses in future evaluations, surrogate models could provide interesting alternatives to reduce computational cost [29].

In evaluating parameter identifiability, structural and practical identifiability are commonly distinguished [27, 28]. This distinguishment is important since practical identifiability may be improved by including more or other measurements [27, 28]. We did not differentiate between structural and practical identifiability since we focused on the use of LV cavity volume and regional strain measurements. However, if future studies reveal that relevant diagnostic parameters are missing, the set of measurements should be extended.

The use of Monte Carlo techniques to calculate diaphony was previously shown to be applicable for relatively large parameter sets [17]. While Van Osta et al. [17] applied this methodology to a CircAdapt configuration with five ventricular wall segments, in the current study, seven or nineteen ventricular wall segments were included. Hereby, the cost function was dominated by the strain and strain rate components (Eqs. 1, 11). Our sensitivity analysis showed, however, that certain model parameters such as $q0$ were particularly sensitive to the volume components. We, therefore, also calculated diaphony for each individual component of the cost function to assess parameter identifiability.

Patients with LV mechanical discoordination are generally associated with reduced pump function resulting from underlying myocardial tissue abnormalities. Our final parameter subset (par-75) included global LV pump function parameters as well as parameters determining regional myocardial activation and constitutive properties and, therefore, seems to include a relevant set of parameters also to describe the pathophysiology of mechanical discoordination. In the current patient population, which was limited in size, we identified relatively consistent patterns of estimated parameter values associated with the underlying pathologies of LBBB and MI. In DTs of LBBB patients, mechanical activation was on average later in the LV free wall than in the septum, while in DTs of MI patients a selection of myocardial segments with increased LGE had a reduced zero-passive stress length, representing a reduced contractility, or an increased stiffness coefficient. As expected, we observed that mechanical activation delay was non-identifiable in hypocontractile segments, which complicated the interpretation of segmental activation delay in most DTs. At the same time, the population size was too small to link abnormal constitutive behavior of the myocardium to increased LGE.

Future studies could investigate whether the parameters included in par-75 sufficiently represent the underlying myocardial tissue properties of the patient. This validation of parameter values is especially important since the framework of parameter subset reduction produces a single subset of reproducible parameters. While this parameter subset is the optimal solution of this framework, it may not be the only reproducible subset that allows generation of patient-resembling DTs based on the integrated measurement information. As part of this validation, parameter estimation in healthy control subjects could be performed.

Credibility of DTs generated using par-75 was supported by regional myocardial work which provides a more integrative measure than individual model parameters. Together, estimated parameter values define the myocardium’s active and passive stress–strain relations which determine regional myocardial work. When calculated in experimental or clinical studies, work is often approximated using non-invasive pressure measurements [30]. These DTs provide a potential advantage here, since they include myocardial stress calculations which allow quantifying work as the enclosed area of the myocardial stress–strain loop.

In agreement with experimental findings [31], our DTs of LBBB-only patients demonstrated a septal-to-lateral imbalance of workload, which was not typically observed in our DTs of MI-only patients (Fig. 4). This observation supports the credibility of our DTs generated. However, while it may have been expected that septal-to-lateral workload imbalance was lower in the LBBB-MI DTs as compared to the LBBB-only DTs [10], no significant difference between these subgroups was found (Fig. 5). We note that our subgroup sizes were currently limited and that statistical significance may have been different when more patients would have been included.

Although in our study, there was no consistent spatial match between regions of increased LGE in the patient and reduced LV myocardial work in the DT, our DTs did show reduced myocardial work in infarcted regions (Fig. 4). Clinical studies using strain measurements to detect non-transmural and transmural infarction have found variable correlations between infarct transmurality and LGE, even in the absence of LBBB [32, 33]. The non-consistent match between reduced myocardial work and increased LGE in our study may, therefore, be considered in line with previous findings.

Comparing our results with other personalized modeling studies in patients with LV mechanical discoordination, Owashi et al. [34] similarly included HFrEF patients with LBBB, and were able to reproduce the measured strain patterns by fitting a similar set of myocardial tissue parameters as included in our final subset. Mineroff et al. [35] additionally personalized parameters determining systemic and pulmonary arterial dimensions as well as valvular dimensions. However, to be able to, they included additional measurements which increased the identifiability of these parameters in their study. As mentioned before, future studies could investigate whether our parameter subset is extensive enough to support clinical decision making or that measurements should be added to identify relevant parameters which are currently missing.

Computational cost presented limitations to the methodologies used in this study. For the initial parameter set, we considered the number of parameter interactions in the 18-segment LV model too high to perform MSM within a feasible time frame. Therefore, we used a 6-segment LV model as a computationally simpler alternative. Following the same reasoning, we also performed Sobol sampling in a 6-segment LV model. Evaluation of three million Sobol simulations took approximately 24 h. A higher-dimensional space as associated with the 18-segment model would have required an exponential increase of the number of simulations to obtain a similar sampling density. We expect that the sensitivity and identifiability of parameters within the 6-segment LV model did not significantly differ from that in the 18-segment model, but we note that this assumption was not verified. Furthermore, in PE, a maximum of five repeated optimization protocols were performed, while a larger number of optimizations would have further improved accuracy of ${\text{ICC}}$ estimations. Repeated optimizations started with a different random seed and ran independent of each other in parallel. Each DMS–PSO evaluation took between 18 and 36 h on a single core, which limited the feasibility of performing more repetitions. During the first iterations of subset reduction, moreover, multiple parameters were removed simultaneously. Reducing per single parameter during all iterations would have been optimal since removing one parameter could increase identifiability of other parameters. By performing DMS–PSO during each iteration, however, we did assess the effect of a simultaneous removal of parameters on ${\chi }^{2}$ so that important parameters were not accidentally removed.

While DMS–PSO is an effective optimization algorithm, it yields a single point estimate of parameter values. Future studies could investigate whether other optimization algorithms based on e.g. Bayesian inference [36] could be used to obtain parameter distributions, which can have important implications when using the DT to support clinical decision making.

To aid in clinical decision making, the DT provides several potential strategies. One strategy is to directly use estimated parameter values as diagnostic or prognostic indices [37]. Second, since the DT is integrated into a model of the heart and circulation, many other indices representing the hemodynamic state of the patient can be derived from the DT and potentially be used as markers of interest. The septal-to-lateral workload imbalance which was quantified in this study is an example of such a derived index and has been used as a marker to predict the effect of pacemaker therapy [38]. Third, the DT may be used for in silico therapy testing, thereby simulating the effect of an intervention. An example would be to simulate pacing therapy in dyssynchronous heart failure patients considered for CRT [39].

## Conclusions

By applying sensitivity and identifiability analysis, we successfully determined a parameter subset of the CircAdapt model which can be used to generate imaging-based digital twins of patients with LV mechanical discoordination. The subset included cardiac output, global LV activation duration, regional mechanical activation delay, and regional LV myocardial constitutive properties. In all patients, these parameters were reproducibly estimated using particle swarm optimization. The digital twin-derived LV myocardial work distribution seemed to provide insight into the patient’s underlying myocardial disease substrate. This DT technology may be used for automatic substrate characterization of patients with LV mechanical discoordination, while future studies should investigate the potential of these DTs to support clinical decision making.

## Methods

### Patient data

#### Patient cohort

A total of 13 patients were retrospectively included. Seven patients with heart failure with reduced ejection fraction (HFrEF) and LBBB were recruited at Maastricht University Medical Center (MUMC), all having LVEF < 35% and QRS width ≥ 130 ms. Patients were selected based on availability of late gadolinium enhancement (LGE) cardiac magnetic resonance (CMR) exams, thereby including four non-ischemic patients (LBBB-only) and three patients with prior MI (LBBB-MI). Patients were furthermore selected based on having good quality of echocardiographic images as assessed by an experienced cardiologist. Furthermore, six post-MI patients with narrow QRS (< 120 ms) (MI-only) were selected from the DEFI-MI (DEtection of Cardiac FIbrosis by LGE–MRI and circulating biomarkers in patients with Myocardial Infarction) cohort, which was established at University Medical Center Utrecht (UMCU) [40] and which included patients with first-time MI. To include patients with sufficient myocardial dysfunction, we selected those DEFI-MI patients with a minimum LV infarct size of 10% of LV wall mass and a minimum LGE transmurality of 25% in at least one LV myocardial segment [12]. The clinical characteristics of these patients are summarized in Table 2.

**Table 2:** Clinical characteristics of the patient population and subpopulations

	All patients (*n* = 13)	LBBB-only (*n* = 4)	MI-only (*n* = 6)	LBBB-MI (*n* = 3)
Age (years)	62 ± 11	61 ± 13	58 ± 10	70 ± 6
Male gender (%, *n*)	85, 11	100, 4	83, 5	67, 2
QRS width (ms)	124 ± 33	153 ± 11	92 ± 13	150 ± 5
LVEF (%)	41 ± 11	31 ± 6	50 ± 3	37 ± 9
LVEDV (%)	134 ± 52	186 ± 34	98 ± 26	139 ± 55

Values are mean ± SD

*LBBB* left bundle branch block, *MI* myocardial infarction, *LVEF* left ventricular ejection fraction, *LVEDV* left ventricular end-diastolic volume

#### Echocardiography

All patients had an echocardiographic exam in the context of the corresponding study where all images were digitally stored and analyzed offline. LBBB-only and LBBB-MI patients were scanned before cardiac resynchronization therapy device implantation using the EPIQ 7 ultrasound system (Philips Medical Systems, Best, The Netherlands). MI-only patients were scanned 6 months after the MI event using a Vivid E9 Ultrasound system (GE Healthcare, Horten, Norway). LV end-diastolic and end-systolic volumes were reassessed by an experienced cardiologist using biplane Simpson’s method (Table 2).

#### Cardiac magnetic resonance

All patients underwent CMR with 2D LGE imaging for visualization of myocardial infarct tissue. All exams were performed on a 1.5 T clinical MR system (Ingenia; Philips Healthcare, Best, The Netherlands). Images were reviewed at the MUMC on a dedicated workstation (Sectra IDS7, Linköping, Sweden), and segmental LGE transmurality was determined by visual assessment of an experienced cardiologist. At the UMCU, images were analyzed offline using Philips ISP9 software (Philips Healthcare, Best, The Netherlands). Using the RV insertion points to the interventricular septum as anatomical landmarks, the heart was subdivided into 16 segments according to the model of the American Heart Association (AHA) [41], excluding the apical cap. The LGE was quantitatively assessed using the full width at half maximum (FWHM) method, providing a percentage for each of the analyzed segments and the total infarct size (global %) of the whole LV.

#### Myocardial strain imaging

Using QLAB advanced quantification software 13 for Philips ultrasound systems (MUMC) or EchoPac version 201 for GE (UMCU), good quality two-chamber, three-chamber and four-chamber echocardiographic acquisitions were used for speckle tracking to obtain 18-segment LV longitudinal strain. Regions of interest were automatically tracked and manually adjusted to both the endo- and epicardial border following the standard recommendations [42]. The first frame in which the mitral valve was closed was manually selected as zero-strain reference.

### Computational model: the CircAdapt model of the human heart and circulation

The CircAdapt model of the human heart and circulation is a closed-loop lumped-parameter model which simulates beat-to-beat hemodynamics and mechanics of the heart and blood vessels [11]. The pulmonary and systemic circulation are modeled using a three-element model of resistive wave impedance, compliance and peripheral resistance [43]. Cardiac walls are modelled as spherical shells, and the left and right ventricular walls are coupled through the interventricular septum using the TriSeg module [44]. Simulation of myocardial active and passive tissue mechanics is based on the three-element Hill contraction model. When walls have homogeneous tissue properties, the model includes a total number of $D=100$ parameters eligible for personalization. These parameters describe global hemodynamics (${D}_{{\text{GH}}}=3$), pulmonary and systemic vessels (${D}_{{\text{PS}}}=12$), valves (${D}_{{\text{V}}}=8$), pericardium (${D}_{{\text{P}}}=3$), mechanical activation (${D}_{{\text{MA}}}=4$), and myocardial tissue properties (${D}_{{\text{MT}}}=70$). Myocardial tissue properties are subdivided over the septum and left ventricular free wall (${D}_{{\text{MT}},{\text{LV}}}=28$), right ventricle (${D}_{{\text{MT}},{\text{RV}}}=14$), left atrium (${D}_{{\text{MT}},{\text{LA}}}=14$), and right atrium (${D}_{{\text{MT}},{\text{RA}}}=14$).

#### 18-segment and 6-segment LV model

Walls are subdivided into different segments to simulate heterogeneity of myocardial tissue properties within the walls [13]. To match the spatial scale of the available echocardiographic strain measurements, an 18-segment LV model was used for parameter estimation in this study. To reduce computational cost during sensitivity and identifiability analysis, however, a simplified 6-segment LV model was used.

Using the 6- or 18-segment LV model influences the total number of parameters. The number of LV myocardial tissue parameters ${D}_{{\text{MT}},{\text{LV}}}$ increases from 28 in the TriSeg model to 14·${n}_{{\text{seg}}}$, with ${n}_{{\text{seg}}}$ the number of wall segments in the septum and LV free wall. In addition, a mechanical activation parameter is added for each extra segment. These changes lead to a total number of $D=160$ parameters and $D=340$ parameters in the 6- and 18-segment model, respectively.

#### Global LV myocardial tissue properties

We hypothesized that LV parameters which were regionally non-identifiable were potentially identifiable on a global LV scale. To check the sensitivity and identifiability of these global LV parameters, we defined these as global offset (GO) parameters, meaning a global reference value for all LV segments. Adding these GO parameters increased the initial number of parameters for the 6-segment model to $D=174$ (Additional file 3: Table S2).

### Framework for parameter subset reduction

**Fig. 6:**
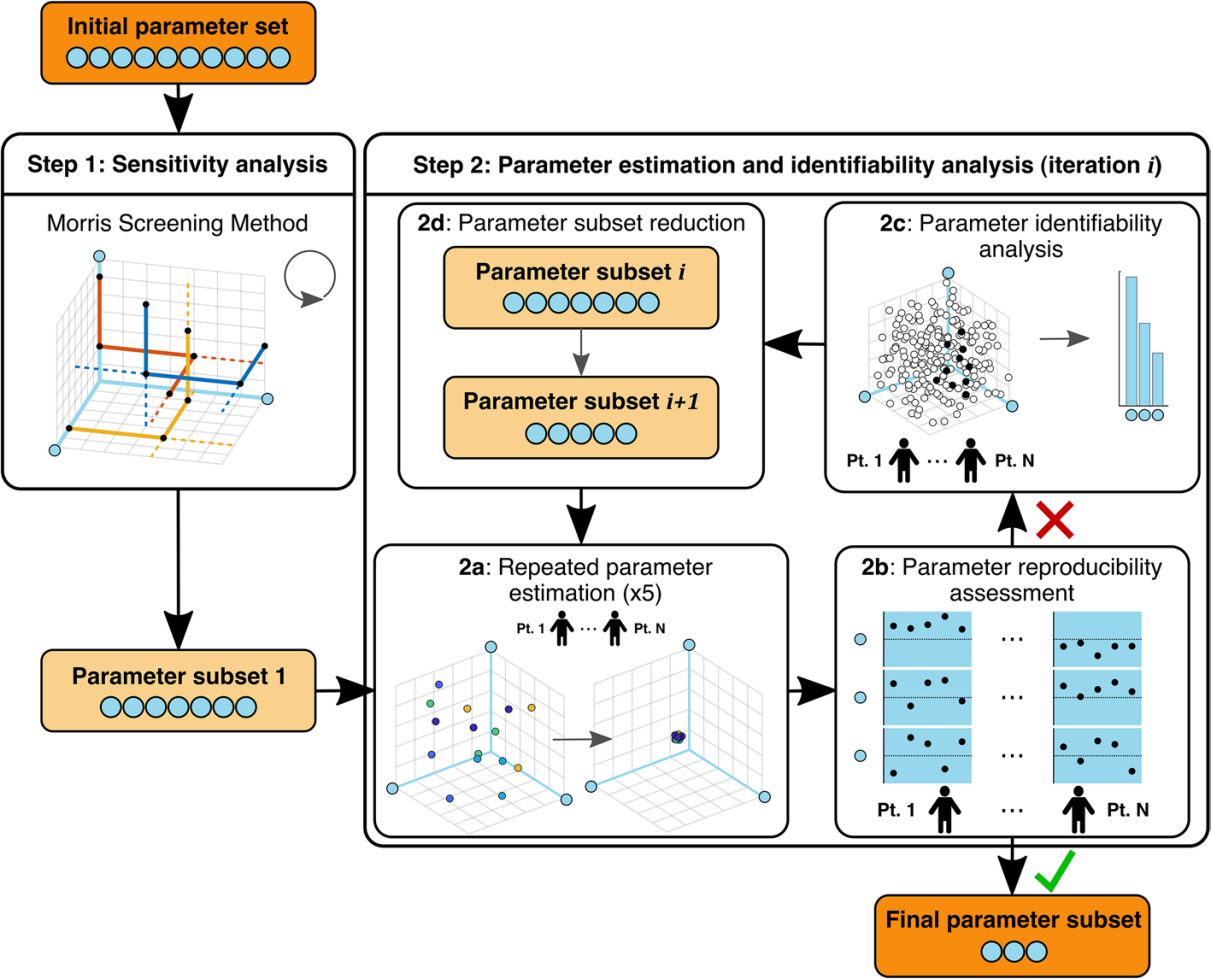
Framework for parameter subset reduction used, based on previous work by van Osta et al. [17]. The framework includes a two-step approach. In Step 1, sensitivity analysis using Morris Screening Method is performed iteratively to find parameters which are important for the model output of interest. In Step 2, using patient-specific measurements, five repeated evaluations of parameter estimation (Step 2a) are performed to assess parameter reproducibility (Step 2b), after which, in case of insufficient parameter reproducibility, identifiability analysis is performed (Step 2c) to further reduce the parameter subset (Step 2d)

The framework for parameter subset reduction used in this study has been introduced elsewhere [17]. The framework includes a two-step approach (Fig. 6), consisting of sensitivity analysis (SA) followed by a combination of PE and identifiability analysis (IA). This section briefly describes the framework and elaborates on a few extensions to the previously published framework.

The methodology used for SA is the Morris Screening Method (MSM) [16, 18] (Step 1), which is a suitable method for models with many parameters. MSM ranks model parameters in order of sensitivity to the model output of interest, represented by the absolute average elementary effect ${\mu }^{*}$. During each iteration of MSM, parameters with below-average ${\mu }^{*}$ for all outputs of interest are removed from the subset (i.e., fixed to their reference value). MSM iterations are repeated until all parameters in the subset have equal to- or above-average ${\mu }^{*}$ for at least one output of interest.

In the second step, the parameter subset is iteratively reduced based on parameter identifiability. To first evaluate identifiability of the current subset, five independent DTs are generated for each patient using dynamic multi-swarm–particle swarm optimization (DMS–PSO) [45] (Step 2a). Reproducibility of parameter estimations is then assessed by calculating the intraclass correlation coefficient (${\text{ICC}}$) (Step 2b). In case of insufficient reproducibility, parameter identifiability is more precisely quantified by Sobol low-discrepancy sampling-derived diaphony (Step 2c). Finally, based on ${\text{ICC}}$ and diaphony, a reduced parameter subset is proposed (Step 2d). This iterative process was stopped when all parameters were identifiable as based on reproducible parameter estimations.

Given the sampling range used for each parameter, the diaphony indicates on a 0-to-1 scale to what extent a parameter has a preferred value within this range. A diaphony close to 1 means that the parameter has a high preference and is likely identifiable. Parameters with the lowest diaphony are, therefore, removed from the subset (Step 2d).

The current study expanded upon the existing framework by not only selecting the samples used for calculating diaphony based on the total cost function value, but also based on the individual components of the cost function. Moreover, the number of parameters removed within one iteration was chosen to depend on the size of the subset. When this subset size was below a certain threshold, in addition, the effect on the value of the cost function was compared between removing a parameter with the lowest diaphony or ${\text{ICC}}$. The following paragraphs will discuss the methodologies of the framework in more detail.

#### Sensitivity analysis

##### Input space

The input space $\Omega ={\mathbb{R}}^{D}$ used for MSM was initially defined by the uncertainty ranges of all $D=174$ parameters of the 6-segment LV model (Additional file 3: Table S2). These ranges aimed to include a wide spectrum of cardiovascular abnormalities which could be found in patients with LV mechanical discoordination.

To simulate relevant pathophysiological activation patterns rather than random activation patterns, a fixed direction of mechanical activation was defined, oriented from the anterior septum (S1) towards the LV posterior wall (LV4). Thereby, mechanical activation ranged from a normal to an LBBB-like activation pattern [7] (Additional file 4: Figure S3). Parameters ${\tau }_{{\text{VV}}}$ and ${\tau }_{{\text{SL}}}$ defined inter- and intraventricular delay, respectively, while parameters ${\alpha }_{1}$, ${\alpha }_{2}$, ${\beta }_{1}$ and ${\beta }_{2}$ determined activation times of the intermediately activated segments.

Furthermore, to simulate the functional consequences of MI, LV active myocardial tissue parameters ${\text{SfAct}}$, ${\text{vMax}}$, ${\text{LDAD}}$, and ${\text{LDCI}}$, and LV passive myocardial tissue parameters ${\text{SfPas}}$ and $k1$ were assigned wider ranges in segments S1, LV1, and LV3 (Additional file 3: Table S2). This definition allowed for a relatively large regional variation of contractile function and stiffness while preserving a minimum degree of LV global contractile function and limiting LV global stiffness.

##### Outputs of interest

**Fig. 7:**
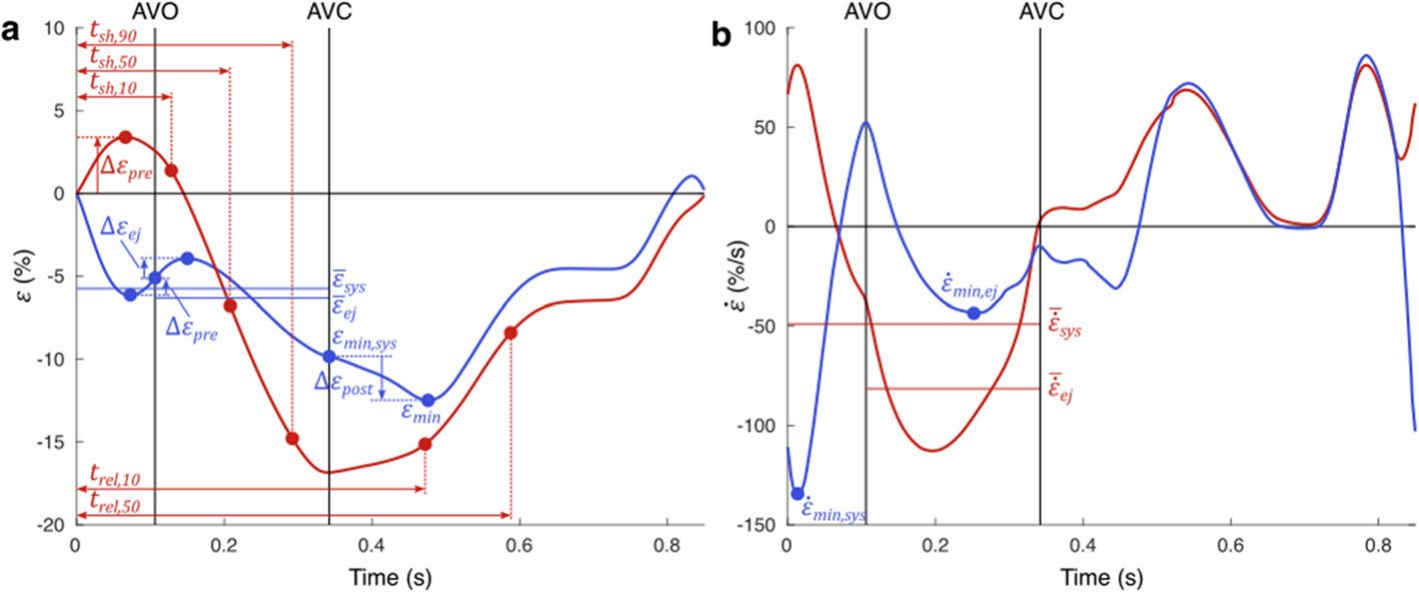
Strain (**a**) and strain rate (**b**) indices calculated as model output in MSM. ${\varepsilon }_{{\text{min}}}$, peak strain; ${\varepsilon }_{{\text{min}},{\text{sys}}}$, peak systolic strain; ${\Delta \varepsilon }_{{\text{post}}}$, post-systolic shortening; $\Delta {\varepsilon }_{{\text{pre}}}$, pre-ejection stretch; $\Delta {\varepsilon }_{{\text{ej}}}$, ejection stretch; ${\overline{\varepsilon }}_{{\text{sys}}}$, mean systolic strain; ${\overline{\varepsilon }}_{{\text{ej}}}$, mean ejection strain; ${\dot{\varepsilon }}_{{\text{min}},{\text{sys}}}$, peak systolic strain rate; ${\dot{\varepsilon }}_{{\text{min}},{\text{ej}}}$, peak ejection strain rate; ${\overline{\dot{\varepsilon }} }_{{\text{sys}}}$, mean systolic strain rate; ${\overline{\dot{\varepsilon }} }_{{\text{ej}}}$, mean ejection strain rate; ${t}_{{\text{sh}},10}$, time to 10% shortening; ${t}_{{\text{sh}},50}$, time to 50% shortening; ${t}_{{\text{sh}},90}$, time to 90% shortening; ${t}_{{\text{rel}},10}$, time to 10% re-lengthening; ${t}_{{\text{rel}},50}$, time to 50% re-lengthening. *AVO* aortic valve opening, *AVC* aortic valve closure

We defined a set of scalar model outputs ${Y}_{j}$ representing the available LV cavity volume and regional strain measurements. The volume outputs included LV end-diastolic volume (EDV) and LV stroke volume (SV). A total of 12 strain indices were calculated for each segment (Fig. 7a): peak strain (${\varepsilon }_{{\text{min}}}$), peak systolic strain (${\varepsilon }_{{\text{min}},{\text{sys}}}$), post-systolic shortening (${\Delta \varepsilon }_{{\text{post}}}$), pre-ejection stretch ($\Delta {\varepsilon }_{{\text{pre}}}$), ejection stretch ($\Delta {\varepsilon }_{{\text{ej}}}$), mean systolic strain (${\overline{\varepsilon }}_{{\text{sys}}}$), mean ejection strain (${\overline{\varepsilon }}_{{\text{ej}}}$), time to 10% shortening (${t}_{{\text{sh}},10}$), time to 50% shortening (${t}_{{\text{sh}},50}$), time to 90% shortening (${t}_{{\text{sh}},90}$), time to 10% re-lengthening (${t}_{{\text{rel}},10}$), and time to 50% re-lengthening (${t}_{{\text{rel}},50}$). Furthermore, 4 regional strain rate indices were calculated (Fig. 7b): peak systolic strain rate (${\dot{\varepsilon }}_{{\text{min}},{\text{sys}}}$), peak ejection strain rate (${\dot{\varepsilon }}_{{\text{min}},{\text{ej}}}$), mean systolic strain rate (${\overline{\dot{\varepsilon }} }_{{\text{sys}}}$), and mean ejection strain rate (${\overline{\dot{\varepsilon }} }_{{\text{ej}}}$). It was assumed that these strain and strain rate indices together sufficiently described the morphology and amplitude of the strain signals.

##### MSM settings

The $D$-dimensional input space was normalized and discretized into a $z$-level grid ($z$ = 8) with normalized parameter values ${x}_{i}\in \left\{0,\frac{1}{z-1},\dots ,1\right\}$ where $i\in \left\{\mathrm{1,2},\dots ,D\right\}$. Each trajectory started at a randomly chosen point on the grid and parameter values were changed one-at-a-time in a random order with step size $\Delta =\frac{1}{2}\frac{z}{z-1}$, such that all points on the grid were equally likely to be included. At each parameterization $x$ the model was evaluated and all outputs ${Y}_{j}$ were calculated. Per iteration we computed a minimum of 1000 successful trajectories, after which we calculated ${\mu }^{*}$ for all input–output relations.

Convergence was checked through a leave-one-out cross-validation, meaning that parameter importance was not changed upon leaving out any of the trajectories. If convergence was not met, an additional 100 successful trajectories were calculated until the leave-one-out condition was satisfied. To limit computational cost, a maximum number of 3000 successful trajectories were calculated, after which confidence intervals were calculated using bootstrapping if convergence was not met. A parameter was then assumed to be non-important for a given output if the 95% confidence interval of ${\mu }^{*}$ was below the average ${\mu }^{*}$ of all parameters.

#### Parameter estimation and identifiability analysis

During PE, model parameters were estimated in the 18-segment LV model to simulate all clinically measured strain signals. Regional LV parameters which were important during MSM in at least one LV segment were estimated for all 18 segments, while important LV GO parameters were estimated only if the parameter was not estimated regionally. This parameter subset was iteratively reduced (Fig. 6, Step 2) to obtain a subset of identifiable parameters.

##### Step 2a: repeated parameter estimation

The initial particle positions for DMS–PSO were determined by generating 1000 Monte Carlo (MC) simulations sampled from a uniform distribution with the same ranges as in MSM, but with additional restrictions on severity of electrical dyssynchrony, global LV contractility and stiffness to prevent unrealistic simulations. The initial particle positions were then defined by the best MC simulations based on the cost function ${\chi }^{2}$, which was defined as the mean squared error:

1 $${\chi }^{2}=\frac{{\chi }_{{V}_{{\text{ED}}}}^{2}+{\chi }_{{\text{EF}}}^{2}+{\chi }_{\varepsilon }^{2}+{\chi }_{\dot{\varepsilon }}^{2}}{2+{n}_{{\text{seg}}}\cdot 2}.$$

With dimensionless contributors:

2 $${\chi }_{{V}_{{\text{ED}}}}^{2}= {\left(\frac{{V}_{{\text{ED}},{\text{mod}}}-{V}_{{\text{ED}},{\text{mea}}}}{{\sigma }_{{V}_{{\text{ED}}}}}\right)}^{2} ,$$

3 $${\chi }_{{\text{EF}}}^{2}= {\left(\frac{{{\text{EF}}}_{{\text{mod}}}-{{\text{EF}}}_{{\text{mea}}}}{{\sigma }_{{\text{EF}}}}\right)}^{2} ,$$

4 $${\chi }_{\varepsilon }^{2}=\sum_{i=1}^{{n}_{{\text{seg}}}}\left(\frac{1}{{n}_{{\text{dp}}}}\sum_{k=1}^{{n}_{{\text{dp}}}}{\left(\frac{{\varepsilon }_{i,{\text{mod}}}(k)-{\varepsilon }_{i,{\text{mea}}}(k)}{{\sigma }_{{\varepsilon }_{i}}}\right)}^{2}\right) ,$$

5 $${\chi }_{\dot{\varepsilon }}^{2}=\sum_{i=1}^{{n}_{{\text{seg}}}}\left(\frac{1}{{n}_{{\text{dp}}}}\sum_{k=1}^{{n}_{{\text{dp}}}}{\left(\frac{{\dot{\varepsilon }}_{i,{\text{mod}}}(k)-{\dot{\varepsilon }}_{i,{\text{mea}}}(k)}{{\sigma }_{\dot{{\varepsilon }_{i}}}}\right)}^{2}\right) .$$

In these equations, ${V}_{{\text{ED}},{\text{mod}}}$ and ${V}_{{\text{ED}},{\text{mea}}}$ are the simulated and measured LV end-diastolic volume, respectively, while ${{\text{EF}}}_{{\text{mod}}}$ and ${{\text{EF}}}_{{\text{mea}}}$ represent the simulated and measured LV ejection fraction. Measurement uncertainties ${\sigma }_{{V}_{{\text{ED}}}}$ and ${\sigma }_{{\text{EF}}}$ were assumed to be proportional to the measured value and equaled $0.13\cdot {V}_{{\text{ED}},{\text{mea}}}$ and $0.14\cdot {{\text{EF}}}_{{\text{mea}}}$, respectively [46]. The number ${n}_{{\text{seg}}}$ equals the number of myocardial segments, while ${n}_{{\text{dp}}}$ is the number of data points used for comparing the simulated and measured strain and strain rate signals. Only strain from mitral valve closure till 10% global re-lengthening + 50 ms was included in the cost function, thereby excluding late diastolic strain. Simulated strain signals ${\varepsilon }_{i,{\text{mod}}}$ were obtained by scaling simulated fiber strain ${\varepsilon }_{i,{\text{mod}},f}$ to the amplitude of the longitudinal strain measurements of the patient:

6 $${\varepsilon }_{i,{\text{mod}},f}\left(t\right)=\frac{{L}_{s,i}\left(t\right)-{L}_{s,i}\left({t}_{0}\right)}{{L}_{s,i}\left({t}_{0}\right)}\cdot 100\%,$$

7 $${\varepsilon }_{i,{\text{mod}}}=\frac{{\varepsilon }_{{\text{glob}},{\text{mea}}}}{{\varepsilon }_{{\text{glob}},{\text{mod}},f}}\cdot {\varepsilon }_{i,{\text{mod}},f.}$$

Here, ${L}_{s,i}(t)$ is the sarcomere length of segment $i$ at time $t$, while ${t}_{0}$ is the timing of mitral valve closure. Furthermore, ${\varepsilon }_{{\text{glob}},{\text{mea}}}$ and ${\varepsilon }_{{\text{glob}},{\text{mod}},f}$ are the measured and simulated peak values of the global strain signal, i.e., the average strain signal of all 18 LV segments. Simulated strains and strain rates were also resampled to the sampling frequency of ${\varepsilon }_{{\text{mea}}}$. Measurement uncertainties ${\sigma }_{\varepsilon }$ and ${\sigma }_{\dot{\varepsilon }}$ were chosen to equal 2% and 20%/s, respectively. Cycle time within the model ($t{\text{Cycle}}$) was fixed to the average cycle time ${t}_{{\text{cycle}}}$ of all three echocardiographic acquisitions. Five independent estimations were obtained by repeating DMS–PSO with different starting points as derived from a new set of MC simulations.

##### DMS–PSO settings

A total of 60 particles were used, subdivided into 20 swarms of three particles. Every 20 iterations, swarms were randomly regrouped. An input space was defined based on the input space used for MC sampling with extended parameter boundaries (Additional file 2: Table S1) to improve algorithm performance. For particles outside these boundaries, the cost function was infinite. Particle velocities were limited to 25% of the input space width to prevent particles from oscillating outside the input space. DMS–PSO was stopped when normalized particle energies were lower than 10^–4^, meaning no parameter changed more than 1% of its MC input space width within one iteration, or when a maximum number of 2000 iterations were completed.

##### Step 2b: parameter reproducibility assessment

Intraclass correlation coefficient (${\text{ICC}}$) was calculated for all estimated parameters by [47]

8 $${\text{ICC}}= \frac{{{\text{MS}}}_{{\text{between}}}-{{\text{MS}}}_{{\text{within}}}}{{{\text{MS}}}_{{\text{between}}}+(m-1)\cdot {{\text{MS}}}_{{\text{within}}}} ,$$

with

9 $${{\text{MS}}}_{{\text{between}}}= \frac{m\cdot {\sum }_{i=1}^{n}{({S}_{i}-\overline{x })}^{2}}{n-1} ,$$

and

10 $${{\text{MS}}}_{{\text{within}}}= \frac{\sum_{i=1}^{n}\sum_{j=1}^{m}{({x}_{ij}-{S}_{i})}^{2}}{n\cdot (m-1)} .$$

In these equations, $n$ is the number of subjects and $m$ equals the number of observations (i.e., repeated optimizations). For a regional LV parameter, $n$ equaled the total number of myocardial segments multiplied by the number of patients, while for a global LV parameter $n$ equaled the number of patients. The term ${x}_{ij}$ represents the $i,j$th component of the $n\times k$ matrix of all data, while ${S}_{i}$ is the mean value for each subject and $\overline{x }$ is the total mean value of all measured values ${x}_{ij}$. The minimum ${\text{ICC}}$ of all parameters in the subset (${{\text{ICC}}}_{{\text{min}}}$) was used as a criterion for accepting or rejecting the current parameter subset. The subset was accepted when ${{\text{ICC}}}_{{\text{min}}}>0.75$, corresponding with good reproducibility [48].

##### Step 2c: parameter identifiability analysis

Using the 6-segment LV model, three million simulations were drawn from a Sobol low-discrepancy sampler using the same parameter ranges as in MSM [49]. Measured strains were averaged in the apex-to-base dimension, and ${n}_{{\text{seg}}}=6$ was used to calculate the strain- and strain rate-based components of the cost function as well as the total cost function value (Eqs. 1, 4, 5). To cover all patients in one set of simulations, $t{\text{Cycle}}$ was added as a parameter to allow for variation in heart rate. For each patient data set $s$, the best ${N}_{{\text{best}}}$ simulations were selected according to ${\chi }_{{\text{IA}}}^{2}$, which was based on the original ${\chi }^{2}$ with an additional term describing deviation of cycle time with doubled weight to increase importance:

11 $${\chi }_{{\text{IA}}}^{2}=\frac{{\chi }_{{V}_{{\text{ED}}}}^{2}+{\chi }_{{\text{EF}}}^{2}+{\chi }_{\varepsilon }^{2}+{\chi }_{\dot{\varepsilon }}^{2}+2\cdot {\chi }_{{t}_{{\text{cycle}}}}^{2}}{4+{n}_{{\text{seg}}}\cdot 2},$$

with:

12 $${\chi }_{{t}_{{\text{cycle}}}}^{2}={\left(\frac{t{\text{Cycle}}-{t}_{{\text{cycle}},{\text{mea}}}}{{\sigma }_{{t}_{{\text{cycle}}}}}\right)}^{2}$$

where ${\sigma }_{{t}_{{\text{cycle}}}}=50 {\text{ms}}$, and $t{\text{Cycle}}$ and ${t}_{{\text{cycle}},{\text{mea}}}$ are the modeled and measured cycle time, respectively. Furthermore, the best ${N}_{{\text{best}}}$ simulations were selected for each individual component of ${\chi }_{{\text{IA}}}^{2}$, meaning ${\chi }_{{V}_{{\text{ED}}}}^{2}$, ${\chi }_{{\text{EF}}}^{2}$, ${\chi }_{{t}_{{\text{cycle}}}}^{2}$, and the strain- and strain rate-based components per segment ${\chi }_{{\varepsilon }_{i}}^{2}$ and ${\chi }_{{\dot{\varepsilon }}_{i}}^{2}$. Diaphony $d$ was then calculated for all parameters $p$ and for all sixteen outputs $q$ per patient data set $s$:

13 $${d}_{p,q}\left(s\right)=\left|\frac{1}{{N}_{{\text{best}}}}\sum_{{x}_{p}\in {X}_{{\text{best}}}\left(s\right)}{e}^{i2\pi {x}_{p}}\right|,$$

with ${X}_{{\text{best}}}(s)$ the set of the best ${N}_{{\text{best}}}=2\cdot {n}_{{\text{par}}}$ samples for data set $s$, with ${n}_{{\text{par}}}$ the number of parameters in the current subset. Parameters $p$ were ranked based on their maximum diaphony over all patient data sets $s$ and outputs $q$. For a regional LV parameter, the maximum diaphony of all six segments was used in this ranking.

##### Step 2d: parameter subset reduction

Initially, parameters with the lowest diaphony were removed from the subset. A regional LV parameter removed from the subset was returned into the subset as a global LV parameter, while global LV parameters removed were fixed to their reference values. Using this reduction approach, regional LV parameters determining the same tissue property in different LV segments were grouped into one parameter group, which was considered to be removed from the subset. When using the term ‘parameter group’ in this section, we, therefore, mean either a non-LV parameter, a global LV parameter, or a group of regional LV parameters. A total of 10, 5 or 3 parameter groups were simultaneously removed from the current subset when more than 30 groups were in the subset, between 16 and 30, or between 11 and 15, respectively. When 10 or fewer parameter groups were in the subset, only one parameter group was removed per iteration. Moreover, two reduced subsets were proposed based on removing the group with lowest diaphony or lowest ${\text{ICC}}$. The removal which caused the lowest maximal increase of ${\chi }^{2}$ out of all patients in PE was chosen as the best option.

### Digital twin credibility evaluation

Experimental models of LBBB have demonstrated an asymmetrical distribution of myocardial work between the septum and LV lateral wall [31]. To evaluate credibility of the DTs generated using the reduced parameter subset, we tested whether the DTs corroborated this observation by calculating a normalized index of septal-to-lateral wall workload imbalance. Specifically, it was hypothesized that this index was higher for both the LBBB-only and LBBB-MI DTs than for the MI-only DTs, while it was also hypothesized that this index was higher for the LBBB-only than for the LBBB-MI DTs [10]. To calculate the index, first, myocardial work ${W}_{i}$ in each of 18 LV segments $i$ was calculated as the area enclosed by the regional stress–strain loop, multiplied with segmental wall volume. Segmental normalized work ${W}_{{\text{norm}},i}$ was then calculated as ${W}_{i}$ normalized by the summed ${W}_{i}$ of all 18 LV segments:

14 $${W}_{{\text{norm}},i}=\frac{{W}_{i}}{\sum_{j=1}^{18}{W}_{j}}$$

Hereafter, septal-to-LV lateral wall workload imbalance ${\Delta W}_{{\text{norm}},\mathrm{ LW}-S}$ was calculated as the difference in summed ${W}_{{\text{norm}},i}$ of the six LV posterolateral wall segments (${\text{LW}}$) and summed ${W}_{{\text{norm}},i}$ of the six septal segments ($S$):

15 $${\Delta W}_{{\text{norm}},{\text{LW}}-S}=\sum_{j\in {\text{LW}}}{W}_{{\text{norm}},j}-\sum_{j\in S}{W}_{{\text{norm}},j}$$

Statistical tests were performed using SPSS Statistics 24 (IBM, Chicago, IL, USA). To test the null hypotheses, the non-parametric Kruskal–Wallis test was performed with post-hoc Bonferroni correction to calculate *p* values.

### Implementation

Equations were linearized using the Newton–Raphson method and were time-integrated using the Adams–Bashford method with a variable timestep $\Delta t$ with max($\Delta t$) = 2 ms. All computations were performed using a C++ implementation of the CircAdapt model as published before [17]. All other codings for SA, PE and IA were performed in MATLAB 2019a (MathWorks, Natick, MA, United States). Simulations were run in parallel on an AMD Ryzen Threadripper 3970X.

### Supplementary Information

**Additional file 1: Figure S1.** Result of the first iteration of Morris Screening Method. Parameters (shown on *x*-axis, numbers corresponding with those in Table S2) were ranked based on their maximum absolute average elementary effect *μ*∗ out of all given outputs of interest (shown on *y*-axis). All parameters which are left of the black vertical line have normalized *μ*∗ > 1 for at least one output of interest and are therefore considered important. **Figure S2.** Result of the final iteration of Morris Screening Method. Parameter numbers shown on the *x*-axis again correspond with those in Table S2. Note that no parameter could be removed based on absolute average elementary effect *μ*∗. Parameters not included in this final iteration were fixed and therefore had zero elementary effect.

**Additional file 2: Table S1.** Parameters included in the different subsets evaluated, and their boundaries used during dynamic multi-swarm particle swarm optimization. Units are the same as in Table S2.

**Additional file 3: Table S2.** All model parameters and ranges used in the Morris Screening Method, based on the 6-segment model.

**Additional file 4: Figure S3.** 6-segment LV model. The blue arrows indicate the sequence of activation as was assumed during Morris Screening Method (MSM) to mimic a left bundle branch block activation pattern. Furthermore, the red segments (S1, LV1, LV3) were assigned wider parameter ranges during MSM to simulate the functional consequences of myocardial infarction.

## Data Availability

The data sets generated and/or analyzed during the current study are available in the Koopsen2024ParameterSubsetReduction repository, https://github.com/CircAdapt/Koopsen2024ParameterSubsetReduction.
