# Supplementary material for: Parameter subset reduction for imaging-based digital twin generation of patients with left ventricular mechanical discoordination
Source: Biomed Eng Online. 2024 May 13;23:46. doi: 10.1186/s12938-024-01232-0 (PMC11089736; doi:10.1186/s12938-024-01232-0)
Supplement: Supplementary file 1 — Additional file 1: Figure S1. Result of the first iteration of Morris Screening Method. Parameters (shown on x-axis, numbers corresponding with those in Table S2) were ranked based on their maximum absolute average elementary effect μ∗ out of all given outputs of interest (shown on y-axis). All parameters which are left of the black vertical line have normalized μ∗ > 1 for at least one output of interest and are therefore considered important. Figure S2. Result of the final iteration of Morris Screening Method. Parameter numbers shown on the x-axis again correspond with those in Table S2. Note that no parameter could be removed based on absolute average elementary effect μ∗. Parameters not included in this final iteration were fixed and therefore had zero elementary effect. [file 12938_2024_1232_MOESM1_ESM.pdf]

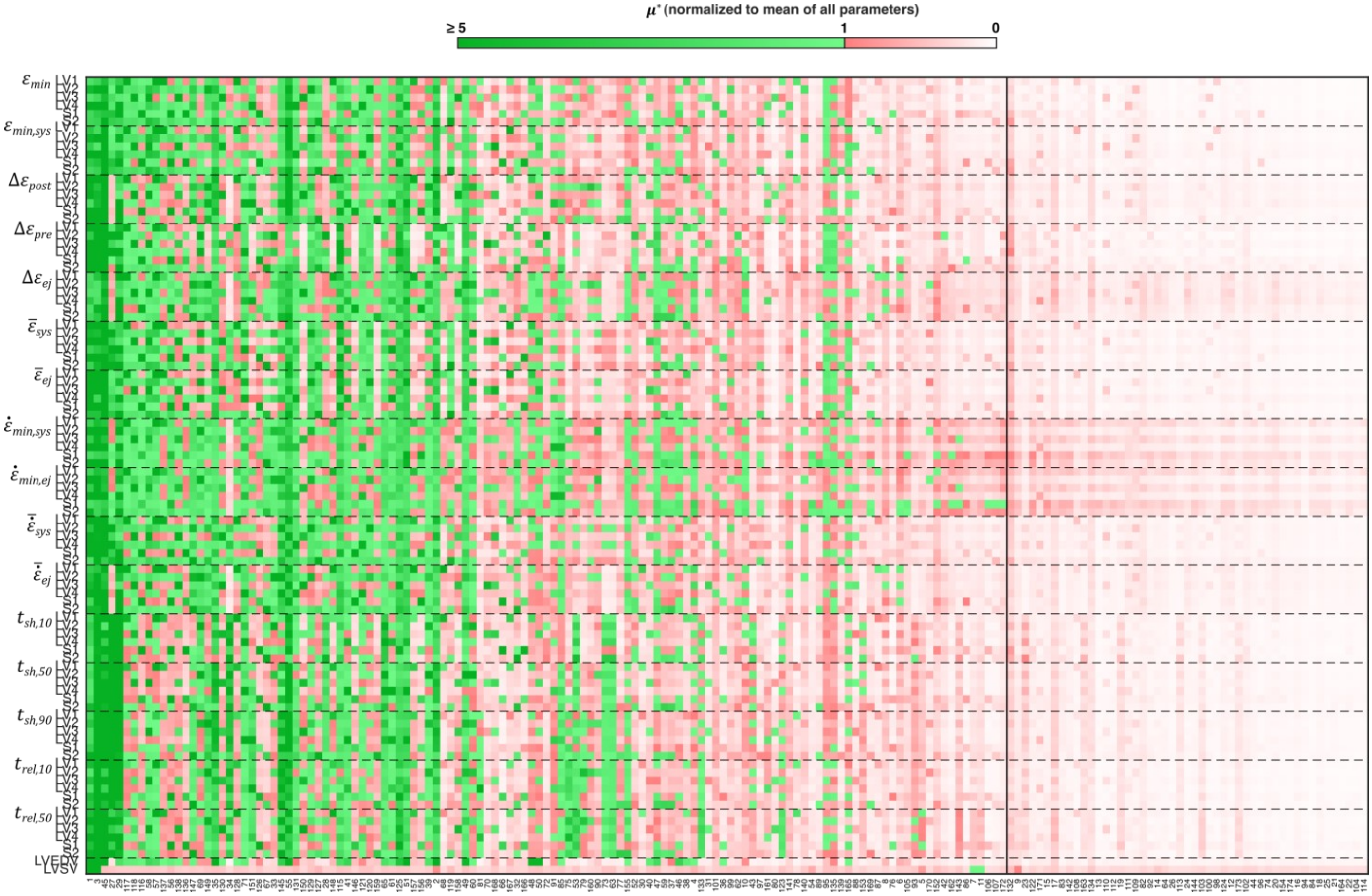

Figure S1: Result of the first iteration of Morris Screening Method. Parameters (shown on x-axis, numbers corresponding with those in **Table S2**) were ranked based on their maximum absolute average elementary effect μ^* out of all given outputs of interest (shown on y-axis). All parameters which are left of the black vertical line have normalized $\mu^* > 1$ for at least one output of interest and are therefore considered important.

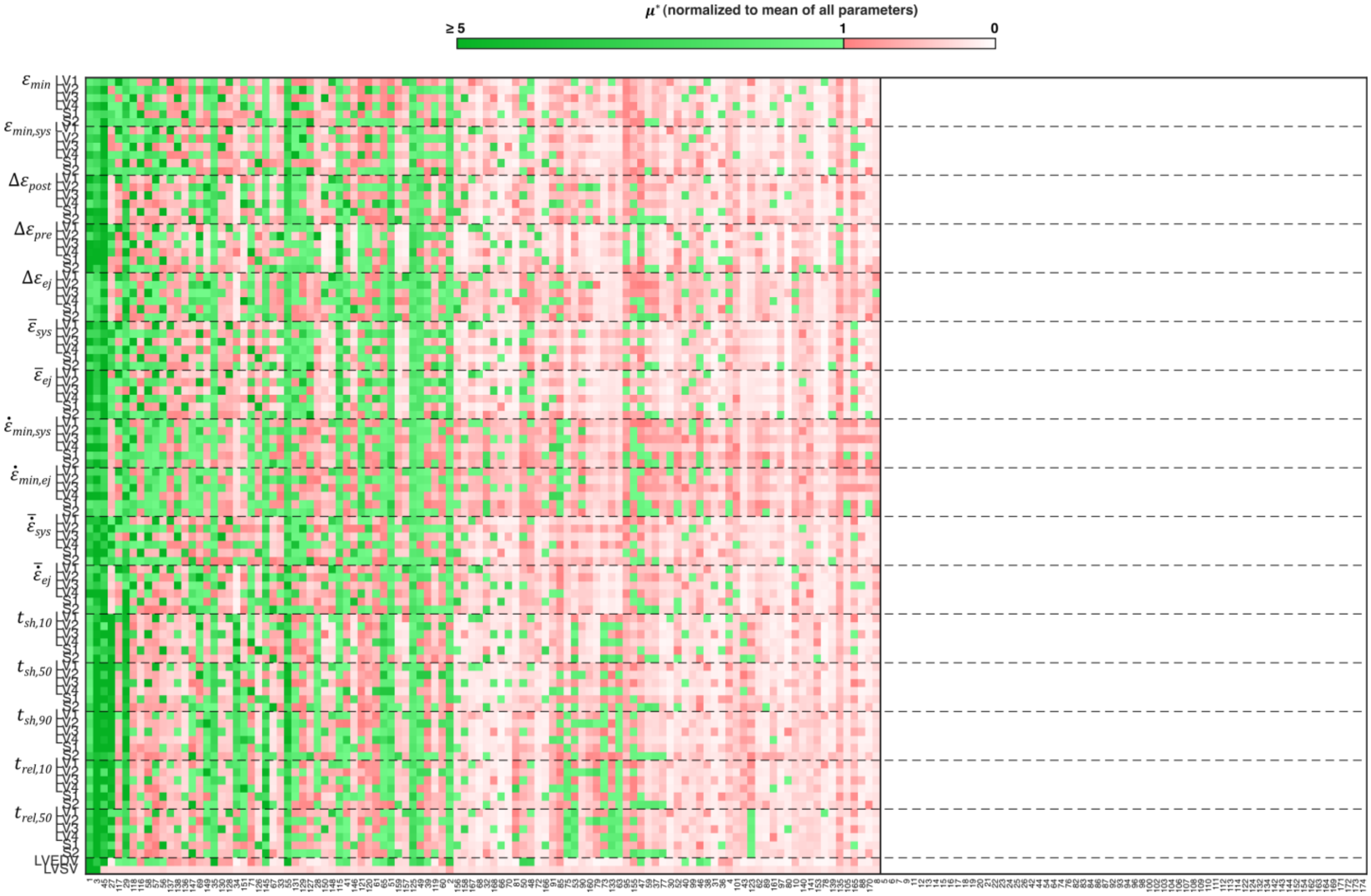

Figure S2: Result of the final iteration of Morris Screening Method. Parameter numbers shown on the x-axis again correspond with those in **Table S2**. Note that no parameter could be removed based on absolute average elementary effect μ^*. Parameters not included in this final iteration were fixed and therefore had zero elementary effect.
