# Supplementary material for: Parameter subset reduction for imaging-based digital twin generation of patients with left ventricular mechanical discoordination
Source: Biomed Eng Online. 2024 May 13;23:46. doi: 10.1186/s12938-024-01232-0 (PMC11089736; doi:10.1186/s12938-024-01232-0)
Supplement: Supplementary file 2 — Additional file 2: Table S1. Parameters included in the different subsets evaluated, and their boundaries used during dynamic multi-swarm particle swarm optimization. Units are the same as in Table S2. [file 12938_2024_1232_MOESM2_ESM.pdf]

Table S1: Parameters included in the different subsets evaluated, and their boundaries used during dynamic multi-swarm particle swarm optimization. Units are the same as in **Table S2**. Abbreviations: Sy, systemic; Seg, segmental; GO, global offset; LA, left atrium; RV, right ventricle; S, septal segments; LV, left ventricular free wall segments.

**Parameter**	**Location**	**par-270**	**par-244**	**par-223**	**par-154**	**par-149**	**par-146**	**par-129**	**par-112**	**par-111**	**par-110**	**par-93**	**par-92**	**par-75**	**par-74**	**Lower bound**	**Upper bound**
$q0$	-	X	X	X	X	X	X	X	X	X	X	X	X	X	X	1	20
$p0$	-	X	X	X	X	X										50	150
k	Sy	X														[0.08, 0.1]	[80, 100]
$p0$	Sy	X														[0.12, 0.001]	[122,1.4]
$A0$	Sy	X														[0.05, 0.05]	[50, 50]
$dTauAv$	-	X	X	X	X	X	X	X	X	X	X	X	X	X	X	-0.100	0.200
dT	Seg	X	X	X	X	X	X	X	X	X	X	X	X	X	X	-0.060 (S)<br>-0.060 (LV)	0.120 (S),<br>0.200 (LV)
	LA	X	X													-0.030	0.150
$VWall$	Seg	X	X	X												1.1 (S)<br>1.6 (LV)	16.2 (S)<br>24.0 (LV)
	GO				X	X										26	385
	LA	X														3	47
$AmRef$	Seg	X	X	X	X	X	X	X	X	X	X	X	X	X	X	1.6 (S)<br>1.6 (LV)	24.4 (S)<br>24.5 (LV)
	GO															29	441
	RV	X	X	X	X											26	389
	LA	X	X													14	208
$SfAct$	Seg	X	X	X	X	X	X									0	1000
	GO							X	X	X						0	1000
	RV	X														0	1000
	LA	X	X	X	X											0	1000
TR	Seg	X	X	X	X	X	X	X								0	1
	GO								X							0	1
	RV	X														0	1
	LA	X														0	1
TD	Seg	X	X	X	X	X	X	X	X	X	X					0	1
	GO											X				0	1
$vMax$	Seg	X	X	X												0.5	50
	GO				X											0.5	50
$dLsPas$	Seg	X														0.01	10
	GO		X													0.01	10
$LenSE$	GO	X														0	0.2
$k1$	Seg	X	X	X	X	X	X	X	X	X	X	X	X	X	X	0	100
	GO															0	100
	LA	X	X	X	X											0	100
$Ls0Pas$	Seg	X	X	X	X	X	X	X	X	X	X	X	X	X	X	0	3
	GO															0	3
	LA	X	X													0	3
$SfPas$	Seg	X	X	X												0	1000
	GO				X	X										0	1000
ADO	Seg	X	X	X	X	X	X	X	X	X	X	X	X			0	2*$t_{cycle,mea}$
	GO													X		0	2*$t_{cycle,mea}$
	LA	X														0	2*$t_{cycle,mea}$
$LDAD$	Seg	X	X													0	2
	GO			X												0	2
$LDCI$	Seg	X	X	X												0	50
	GO				X											0	50
