# Supplementary material for: Parameter subset reduction for imaging-based digital twin generation of patients with left ventricular mechanical discoordination
Source: Biomed Eng Online. 2024 May 13;23:46. doi: 10.1186/s12938-024-01232-0 (PMC11089736; doi:10.1186/s12938-024-01232-0)
Supplement: Supplementary file 3 — Additional file 3: Table S2. All model parameters and ranges used in the Morris Screening Method, based on the 6-segment model. [file 12938_2024_1232_MOESM3_ESM.pdf]

Table S2: All model parameters and ranges used in the Morris Screening Method, based on the 6-segment model. Abbreviations: Sy, systemic; Pu, pulmonary; MV, mitral valve; AV, aortic valve; TV, tricuspid valve; PV, pulmonary valve; GO, global offset; S, septal segments; LV, left ventricular free wall segments; RV, right ventricle; LA, left atrium; RA, right atrium.

	Nr.	Parameter	Location	Physiological meaning	Unit	Lower bound	Upper bound
Global hemodynamics (D$_{GH}$ = 3)	1	$q0$	-	Cardiac output	L/min	2.1	5.5
	2	$p0$	-	Mean arterial pressure	mmHg	69	114
	3	$tCycle$	-	Cycle time	s	0.66	1.24
Arterio-venous system (D$_{AV}$ = 12)	4	k	Sy	Stiffness exponent [inlet, outlet]	-	[6.4, 8.0]	[9.6, 12.0]
	5		Pu				
	6	Len	Sy	Characteristic length [inlet, outlet]	m	[0.32, 0.32]	[0.48, 0.48]
	7		Pu			[0.16, 0.16]	[0.24, 0.24]
	8	$p0$	Sy	Reference working pressure [inlet, outlet]	kPa	[9.7, 0.11]	[14.6, 0.17]
	9		Pu			[2.0, 0.40]	[3.0, 0.61]
	10	$A0$	Sy	Reference cross-sectional area [inlet, outlet]	cm²	[4.0, 4.0]	[6.0, 6.0]
	11		Pu			[3.8, 4.1]	[5.6, 6.2]
	12	$AWall$	Sy	Cross-sectional wall area [inlet, outlet]	cm²	[0.91, 0.37]	[1.37, 0.55]
	13		Pu			[0.71, 0.39]	[1.06, 0.58]
	14	kAV	Sy	Resistance exponent	-	0.8	1.2
	15		Pu			1.6	2.4
Pericardium (D$_P$ = 3)	16	k	-	Stiffness exponent	-	8	12
	17	$VRef$	-	Reference volume	L	0.53	0.80
	18	$pAdapt$	-	Adaptation pressure	mmHg	80	120
Valves (D$_V$ = 8)	19	$AOpen$	MV	Opening area	cm²	6.0	9.0
	20		AV			4.0	6.0
	21		TV			5.6	8.4
	22		PV			3.8	5.6
	23	Len	MV	Characteristic length	m	0.013	0.020
	24		AV				
	25		TV				
	26		PV				
Mechanical activation (D$_{MA}$ = 8)	27	$dTauAv$	-	Atrioventricular delay with respect to reference	s	-0.050	0.130
	28	τ_{VV}	-	Interventricular delay	s	-0.030	0.030
	29	τ_{SL}	-	Maximum intraventricular delay (septum to LV lateral wall)	s	0	0.120
	30	α_1	-	Delay factor LV1	-	1/6	3/6
	31	α_2		Delay factor LV3		3/6	5/6
	32	β_1		Delay factor S2		1/6	3/6
	33	β_2		Delay factor LV2		3/6	5/6
	34	dT	LA	Delay of mechanical activation	s	-0.010	0.050
Myocardial tissue (D$_{MT}$ = 140)	35	$VWall$	GO	Wall volume	mL	103	154
	36-37		S1-2			10	23
	38-41		LV1-4			15	35
	42		RV			50	76
	43		LA			12	19
	44		RA			5.1	7.7
	45	$AmRef$	GO	Reference wall area	cm²	118	176
	46-47		S1-2			16	35
	48-51		LV1-4			16	35
	52		RV			104	156
	53		LA			55	83
	54		RA			48	72
	55	$SfAct$	GO	Active stress constant	kPa	60	144
	56, 57, 58		S1, LV1, LV3			0	173
	59, 60, 61		S2, LV2, LV4			48	173
	62		RV			60	144
	63		LA			42	101
	64		RA				
	65	TR	GO	Contractility rise time constant	s	0.20	0.30
	66-67, 68-71		S1-2, LV1-4			0.16	0.36
	72		RV			0.20	0.30
	73		LA			0.32	0.48
	74		RA				
	75	TD	GO	Contractility decay time constant	s	0.20	0.30
	76-77, 78-81		S1-2, LV1-4			0.16	0.36
	82		RV			0.20	0.30
	83		LA			0.32	0.48

	84		RA				
	85	$vMax$	GO	Maximum sarcomere shortening velocity	µm/s	5.6	8.4
	86, 87, 88		S1, LV1, LV3			2.8	10.1
	89, 90, 91		S2, LV2, LV4			4.5	10.1
	92		RV			5.6	8.4
	93		LA			11.2	16.8
	94		RA				
	95	$dLsPas$	GO	Titin stiffness exponent	-	0.48	0.72
	96-97, 98-101		S1-2, LV1-4			0.38	0.86
	102		RV			0.48	0.72
	103		LA				
	104		RA				
	105	$LenSE$	GO	Series elastic element length	µm	0.032	0.048
	106-107, 108-111		S1-2, LV1-4			0.026	0.058
	112		RV			0.032	0.048
	113		LA				
	114		RA				
	115	$k1$	GO	ECM stiffness exponent	-	8	12
	116, 117, 118		S1, LV1, LV3			6.4	36
	119, 120, 121		S2, LV2, LV4			6.4	14.4
	122		RV			8	12
	123		LA				
	124		RA				
	125	$Ls0Pas$	GO	Zero-passive stress sarcomere length	µm	1.71	1.89
	126-127, 128-131		S1-2, LV1-4			1.63	1.99
	132		RV			1.71	1.89
	133		LA				
	134		RA				
	135	$SfPas$	GO	Passive stress coefficient	kPa	17.8, 17.7*	26.8, 26.5*
	136		S1			14.1	265
	137, 138		LV1, LV3			14.3	268
	139		S2			14.1	31.8
	140, 141		LV2, LV4			14.3	32.1
	142		RV			18.2	27.3
	143		LA			40.8	61.2
	144		RA			42.0	63.0
	145	ADO	GO	Activation duration offset	s	0.52	0.78
	146-147, 148-151		S1-2, LV1-4			0.42	0.94
	152		RV			0.52	0.78
	153		LA				
	154		RA				
	155	$LDAD$	GO	Length dependency of activation duration	s	0.85	1.27
	156, 157, 158		S1, LV1, LV3			0.42	1.52
	159, 160, 161		S2, LV2, LV4			0.68	1.52
	162		RV			0.85	1.27
	163		LA				
	164		RA				
	165	$LDCI$	GO	Length dependency of contractility increase	-	7.3	10.9
	166, 167, 168		S1, LV1, LV3			3.6	13.1
	169, 170, 171		S2, LV2, LV4			5.8	13.1
	172		RV			7.3	10.9
	173		LA				
	174		RA				

* The left ventricular free wall segments (LV) and septal segments (S) have different values for $SfPas$, therefore this number represents the values of LV, S
