# Supplementary material for: Parameter subset reduction for imaging-based digital twin generation of patients with left ventricular mechanical discoordination
Source: Biomed Eng Online. 2024 May 13;23:46. doi: 10.1186/s12938-024-01232-0 (PMC11089736; doi:10.1186/s12938-024-01232-0)
Supplement: Supplementary file 4 — Additional file 4: Figure S3. 6-segment LV model. The blue arrows indicate the sequence of activation as was assumed during Morris Screening Method (MSM) to mimic a left bundle branch block activation pattern. Furthermore, the red segments (S1, LV1, LV3) were assigned wider parameter ranges during MSM to simulate the functional consequences of myocardial infarction. [file 12938_2024_1232_MOESM4_ESM.pdf]

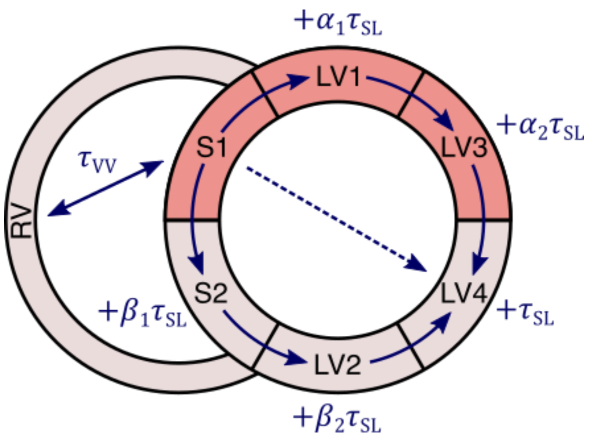

Figure S3: 6-segment LV model. The blue arrows indicate the sequence of activation as was assumed during Morris Screening Method (MSM) to mimic a left bundle branch block activation pattern. Furthermore, the red segments (S1, LV1, LV3) were assigned wider parameter ranges during MSM to simulate the functional consequences of myocardial infarction.
